# Efficacy of Alternative Medicine in Reducing Hemoglobin A1c (HbA1c) in Type 2 Diabetes Mellitus

**DOI:** 10.7759/cureus.10246

**Published:** 2020-09-04

**Authors:** Sagar Kumar, Priyanka Kumari, Vikash Kumar, Maham Fatima

**Affiliations:** 1 Medicine, Jinnah Post Graduate Medical Center, Karachi, PAK; 2 Internal Medicine, Chandka Medical College, Larkana, PAK; 3 Internal Medicine, Jinnah Sindh Medical University, Karachi, PAK

**Keywords:** diabetes mellitus type 2, complementary and alternative medicine, pakistan

## Abstract

Introduction: Diabetes mellitus is one of Pakistan's most common diseases, affecting nearly 27.4 million people. Complementary and alternative medicine (CAM) is becoming quite popular for the management of diabetes. We aim to study the subjects that use alternative medicine in order to assess the effect of alternative therapy on the glycemic control and overall health of the patients.

Methods: This study was conducted from January 2019 to December 2019 in Karachi, Pakistan. A total of 101 diabetic patients who used alternative medicine as part of their diabetes treatment were enrolled in the study after oral informed consent. Patients who were using concomitant conventional therapy were excluded from the study. On day 0, the random blood glucose levels and hemoglobin A1c (HbA1c) levels were noted. Patients were followed up after 12 weeks, and their blood glucose levels and HbA1c levels were noted again. Out of 101 participants, around 92 of them completed the study and were included in the final analysis.

Results: According to the results, 41 (44.5%) participants admitted that they used Herbal medicine, 32 (34.78%) participants used home remedies through dietary modifications, 11 (11.95%) participants used spiritual healing techniques, and 7 (7.60%) participants used cupping therapy. We found no significant reduction of HbA1c levels at the end of 12 weeks in any group.

Conclusion: This study failed to show any significant reduction in HbA1c levels after 12 weeks in patients taking various alternative medicine forms. There is a need for further large-scale trials to establish this mode of treatment's efficacy and safety.

## Introduction

Diabetes mellitus is widely prevalent worldwide, with as many as 415 million people suffering from this disease [1]. It is one of the leading causes of morbidity and mortality. Rank wise, it is the eighth major cause of death in both the genders [2]. Modern treatments have improved the disease course and overall clinical outcome; however, it is a major international public health concern due to the increased prevalence of this disease. Diabetes mellitus is also quite common in Pakistan as Pakistan ranks sixth with the diabetic population percentage. Approximately 26.3% of Pakistan's population, i.e., 27.4 million people, are suffering from diabetes mellitus [3]. The most effective way to control blood glucose levels and prevent diabetes complications is by educating patients regarding dietary modifications, regular exercise, and recommending medications [4].

Apart from the mentioned traditional ways to combat the disease, complementary and alternative medicines (CAM) have also become popular. These CAM are different from traditional therapeutic practices and are not medically proven [5]. Examples of CAM include modified diets, spiritual healing, aromatherapy, herbal medicines, and acupuncture. CAM practices are as common as 18% to 72.8% in nine out of ten countries worldwide [6]. CAM therapy's choice depends on variables, such as educational status, salary, religious values, and affordability [7]. The most accepted types of CAM therapy used are homeopathic medications and herbal therapies [8].

In Karachi, 48% of patients used herbal medicines, while 53.5% of patients used other forms of complementary medicines according to a study [7]. There have been variable data regarding the type of practice being used. According to a study, 46.7% of people reportedly used herbal medicines, 24.3% of patients used spiritual therapy, 16.4% used Unani medications, and 8.4% used homeopathic medicines.

Despite the popularity of CAM as an adjunct therapy for diabetes mellitus in Pakistan, no such study determines its correlation with glycemic control. Therefore, we plan to study the subjects that use alternative therapy to assess the effect of CAM on the patients' glycemic control and overall health. This study will help us improve clinical practice and help us educate general practitioners regarding these non-medical therapies.

## Materials and methods

This study was conducted from January 2019 to December 2019 in Karachi, Pakistan. A total of 101 diabetic patients who used alternative medicine for the treatment of diabetes were enrolled in the study after oral informed consent. Patients who were using concomitant conventional therapy were excluded from the study. We checked patients' random blood glucose levels and hemoglobin A1c (HbA1c) levels at day 0 of our study. Patients were followed up after 12 weeks, and their blood glucose levels and HbA1c levels were noted again. Approximately 92 participants completed the study and were included in the final analysis. Patient's age, gender, type of alternative medicine, blood glucose levels, and HbA1c levels were noted in a self-structured questionnaire.

Data were processed and analyzed using SPSS for Windows, version 23.0 (IBM Corp., Armonk, NY). Mean and standard deviation (SD) were calculated for continuous variables and frequencies. Percentages were calculated for categorical variables. Mean blood glucose levels and HbA1c at day 0 and week 12 were compared using a dependent t-test. A p-value of less than 0.05 indicates that the difference at day 0 and week 12 is significant enough to discard the null hypothesis.

## Results

Out of 92 participants who completed the study, 51 (55.4%) were males and 41 (44.6%) were females. The mean age of participants was 46 ± 6 years. Around 41 (44.5%) participants admitted taking herbal medicines, 32 (34.78%) participants used home remedies through dietary modifications, 11 (11.95%) participants used spiritual healing, and 7 (7.60%) participants used cupping therapy. There were no significant reduction of HbA1c levels at the end of 12 weeks (Table 1).

**Table 1:** Comparison of HbA1c at Day 0 and Week 12 HbA1c, Hemoglobin A1c *Means non-significant

Type of Alternative Medicine	HbA1c(%) at Day 0	HbA1c (%) at Week 12	P Value
Herbal medicine (n = 41)	6.45 ± 1.01	6.32 ± 0.92	0.54*
Treatment based on specific diet (n = 32)	6.52 ± 0.98	6.44 ± 1.00	0.74*
Spiritual healing (n = 11)	6.72 ± 1.11	6.56 ± 1.07	0.73*
Cupping (n = 7)	6.43 ± 0.99	6.40 ± 1.00	0.97*

## Discussion

CAM for the treatment of diabetes is prevalent in Pakistan. In a recent study, almost one-third of patients with diabetes admitted using CAM to treat diabetes [9]. In our study, herbal medicine was the most common form of alternative medicine, followed by home remedies through dietary modifications. This result was contrary to a qualitative analysis from Pakistan, where religious practice (37.5%) was the most common alternative therapy used, followed by the use of herbal therapy (15.5%) [10].

In this study, even though there was a numerical reduction in serum HbA1c level, it was not significant (p-value >0.05). Raja et al. also showed that participants who practiced CAM for diabetes had poor glycemic control [9]. In their 2002 to 2007 National survey conducted in the United Kingdom, Nahin et al. stated that the use of alternative medicine results in poor glycemic control [11]. According to Handley et al., complementary health approach in type 2 diabetes may be associated with poor cardiometabolic control and medication adherence [12]. On the contrary, various studies show the benefit of complementary medicine as well. In a Nigerian study, CAM's use was associated with better glycemic control and an improved lipid profile [13]. Choudhury et al. in a systemic review stated that several herbal plants proved to be beneficial for glycemic control in type 2 diabetes [14]. In an Iranian study, there was a significant positive correlation between self-care activities related to foot care and religious practices [15]. Akbari et al. found that cupping therapy in diabetic patients resulted in a significant reduction in various parameters, such as HbA1c levels and preprandial and postprandial blood sugar levels. There was also a reduction in serum triglyceride, serum cholesterol, and low-density lipoprotein [16].

Various factors influence the use of CAM in diabetes. According to Nahin et al., severe disease and longer diabetes duration are associated with greater use of CAM therapy [11]. A Nigerian study reported safety (74%) and affordability (60%) as the most common reason for use of CAM in type 2 diabetes [15]. They also reported older age, lower educational status, and longer diabetes duration as predictors of herbal medicine use. Similar findings were reported by Raja et al., who stated that the use of CAM is associated with older age, lower education status, longer duration of diabetes, female gender, and poor glycemic control. They also also reported other reasons, such as lack of trust in pharmaceutical products, longer waiting intervals to see physicians, and poor patient-doctor relationship to start CAM [9].

To the best of our knowledge, this is the first study from Pakistan that studies the effect of complementary medicine on HbA1c levels in type 2 diabetes. The use of CAM for diabetes is becoming prevalent in Pakistan. However, there is minimal data available on the safety and efficacy of this mode of treatment. There should be a combined effort of clinicians and researchers to initiate safety and efficacy trials on common alternative and complementary therapy for diabetes treatment.

## Conclusions

This study showed no significant reduction in the serum HbA1C levels after 12 weeks in patients taking various forms of alternative medicine. In this study, herbal medicine was the most common alternative therapy, followed by home remedies through dietary modification, spiritual healing, and cupping. There is a need for further large-scale trials to establish the efficacy and safety of this mode of treatment. 
